# Greater Postural Sway and Tremor during Balance Tasks in Patients with Plantar Fasciitis Compared to Age-Matched Controls

**DOI:** 10.3390/healthcare8030219

**Published:** 2020-07-20

**Authors:** Jerrold Petrofsky, Robert Donatelli, Michael Laymon, Haneul Lee

**Affiliations:** 1 School of Physical Therapy, Touro University Nevada, Henderson, NV 89002, USA; Jerrold.Petrofsky@tun.touro.edu (J.P.); Michael.Laymon@tun.touro.edu (M.L.); 2 Modern Athletic Science, Las Vegas, NV 88901, USA; bobbyd1950@gmail.com; 3 Department of Physical Therapy, Gachon University, Incheon 400011, Korea

**Keywords:** foot, balance, posture sway, pain, plantar fasciitis

## Abstract

Plantar fasciitis (PF) is a common condition found in men and women and can reoccur throughout life. PF is commonly diagnosed by prolonged foot pain lasting more than 3 months and a plantar fascia over 0.4 mm thick, as measured using ultrasound imaging. This study examined the ability to balance and the occurrence of muscle tremor during different balance tasks in patients with PF compared to their control counterparts. Fifty subjects (25 patients with PF and 25 control subjects) participated in this study. Subjective pain (measured with a visual analog scale (VAS)), pressure pain threshold (PPT), and postural sway and tremor during eight different balance tasks were measured. Postural sway was measured by a balance platform, while tremor was measured as the mechanical movement of the platform in the 8 Hz frequency range. Thickness of plantar fascia, subjective pain, and PPT were significantly greater in the PF group compared to the controls (*p* < 0.001). Postural sway and 8 Hz tremor were significantly greater in the PF group compared to the control group for all eight balance tasks (*p* < 0.01). These results indicate that the lack of plantar fascia elasticity is probably the cause of the reduced balance and increased muscle tremor.

## 1. Introduction

While most muscles in the calf insert on the calcaneus, power is transferred across the foot and to the toes by a thick band of tissue that originates at the medial tuberosity of the calcaneus and inserts into the proximal phalanges [1]. This tissue, the plantar fascia, is divided into five fascicles, one extending to each of the toes [2]. The central part of the plantar fascia is attached to the posterior aspect of the medial tuberosity of the calcaneus, posterior to the origin of the flexor digitorum brevis tendon. While the width here is between 1.5 and 2 cm, the thickness is usually less than 0.5 cm. The plantar fascia can become inflamed at any time in life [3,4]; this is commonly due to age, high body mass index (BMI), and overuse such as might occur in athletes [5,6]. There are numerous treatment modalities that have been used to treat plantar fasciitis (PF), but the condition generally returns during life and can impair gait [7]. While pain is indicative of PF, the thickness, when measured 0.5 cm from the calcaneus insertion of the plantar fascia, is normally 0.4 mm or less in healthy individuals and greater than 0.4 mm in individuals with PF [8,9].

The mechanical function of the plantar fascia is to act as an elastic spring to cushion the foot and to transfer energy from the ankle flexors and extensors to the toes for balance and gait. Like other tendons and ligaments, its elasticity and hence function varies in normal daily activities and disease. People with a less elastic fascial tissue get strained more easily under loading [10]. In women, for example, the plantar fascia has more elasticity during ovulation due to higher estradiol level, while it thins and gains elasticity near menses [11,12]. This has an effect on foot lengthening during weight bearing. In women with a normal menstrual cycle, the foot lengthens more during ovulation in proportion to the weight applied to it, compared to menstruation where the increased strength in the plantar fascia causes less lengthening in response to load (an increase in elastic strength) [11,12]. This change in elasticity has an effect on balance. When a series of balance tasks were accomplished during these same two periods of the menstrual cycle, balance (static) was worse at ovulation when the plantar fascia was less elastic.

Tremor is an error in motor control. It is caused by the time delay from movement activating the muscle spindles and the sensory output traveling to the spinal cord and altering muscle activity through the motor nerve. It is a monosynaptic reflex and requires about one-eighth of a second in the average person. If there is a time delay from the time the spindles receive a change in muscle tension due to laxness in tendons or ligaments, then the muscle would respond slower and increase motor error (tremor). Thus, anything that allows for laxness in a tendon or ligament should increase motor error [13,14]. Further, when tremor was measured in the 8 Hz bandwidth from the balance platform data, tremor increased significantly at ovulation [11,12,15]. Thus, reduced elasticity probably caused delayed transfer of mechanical energy through the foot from the calcaneus, causing motor errors and poor balance at ovulation compared to menstruation. The ankle has more laxness at ovulation compared to other periods of the menstrual cycle [15,16]. In another study on women during the menstrual cycle, changes in mechanical properties of the ankle muscles such as tone, stiffness, and elasticity were correlated to postural sway [11,17,18].

Other studies on runners show that an increase in thickness and water content of the plantar fascia was also seen, and that it also caused a reduction in elasticity [19]. Studies on PF also show an increase in thickness of the plantar fascia, which then caused a reduction in plantar fascia elasticity [20,21,22]. As was seen for the menstrual cycle, this reduction in elasticity likewise should show an increase in balance and tremor on the balance platform. However, the effect of PF on postural sway and its tremor during different balance tasks has not been assessed yet. Thus, the present investigation tested the hypothesis that chronic PF would reduce balance and motor control.

## 2. Methods

### 2.1. Subjects

Fifty individuals with PF and healthy counterparts between the ages of 30 to 70 years old participated in this study (25 individuals with PF and 25 age-matched healthy counterparts). PF subjects were included if they had received a primary clinical diagnosis of PF with diagnostic sonography, and controls were healthy counterparts. Subjects were excluded if they had been diagnosed as having systemic arthritis, lupus, scleroderma, multiple sclerosis, or other metabolic diseases, as were subjects who had suffered a recent acute trauma. Subjects taking any medications that could alter balance and subjects with vision/vestibular problems or orthopedic/neuromuscular/cardiovascular impairments were also excluded from the study. General characteristics of the subjects are described in Table 1.

### 2.2. Procedures

This study was approved by the Salus Institutional Review Board (Salus-31620). All subjects signed an informed consent form before study participation. Once participants agreed to participate in the study, the general characteristics of the subjects were recorded. Then, subjective pain and pressure pain threshold (PPT) were assessed, and plantar fascia thickness was measured by musculoskeletal ultrasound (US). After the measurements, the investigator briefed the participants about the balance tasks. The participants were given sufficient time to familiarize themselves with the tasks and to practice once. Then, postural sway and tremor were recorded during the eight different balance tasks.

### 2.3. Outcome Measurement

#### 2.3.1. Plantar Fascia Thickness

Plantar fascia thickness was assessed at the calcaneal insertion, 5 mm distal from the medial calcaneal tuberosity, with a Sonosite MicroMaxx Ultrasound System with L35 −10 MHz linear probe (Fujifilm Sonosite, Bothell, WA, USA). All US measurements were taken by an experienced musculoskeletal sonographer with over 20 years of experience.

The basis for the ultrasound measurement of plantar fascia thickness for PF diagnosis has been published recently in a review article. Numerous studies have examined the applicability of ultrasound imaging to the diagnosis of PF. In a recent of review of 34 articles, it was found that ultrasound imaging was reliable in repeatedly measuring plantar fascia thickness and the results of interventions on plantar fascia thickness [8]. Further, plantar fascia thickness was directly correlated to pain and vascularity of the plantar fascia [23].

#### 2.3.2. Subjective Pain

Subjective pain was assessed through a visual analog scale (VAS). A 10 cm VAS is commonly used as a subjective measure of pain [24,25,26]. It uses a horizontal line across a piece of paper. The line is 10 cm long [27,28]. One end is marked “pain free” and the other end is marked “very, very sore”. The subject was asked to place a vertical slash across the line where appropriate. The reliability and validity of this scale have been tested extensively and found to be moderately valid compared to other pain measures in many studies [29,30].

#### 2.3.3. Pressure Pain Threshold (PPT)

The PPT, minimum pressure or force producing pain, was measured with a handheld pressure algometer (Wagner Inc., Greenwich Conn, CT, USA). The pressure algometer is a force gauge fitted with a tip and calibrated in newtons (N). With the patient in a supine position with legs fully extended, the tender point (measuring point) on the plantar fascia was established with reference to anatomical landmarks and marked with measuring tape. Then, the investigator gradually increased the pressure until the subject reported pain, with the pressure algometer aligned perpendicularly to the skin. The excellent reliability and validity of a pressure algometer as a measure of pain has been reported in previous studies [31,32,33].

#### 2.3.4. Balance Measurements

There were eight different tests that were used in this study. The tests were selected at random order and represented different levels of challenge to balance. The subjects were asked to either balance under loss of one, two, or three factors that influence the ability to balance. The first factor was either surface compliance or vision, the second factor was base of support or vision, and the third factor that could be removed was to alter surface compliance and vision and base of support. The eight tests and factors altered were as follows [34]:

1. FAEO firm—feet apart, eyes open, firm base of support;
2. TEO firm—feet tandem, eyes open, firm base of support;
3. FAEO foam— feet apart, eyes open, foam;
4. FAEC firm—feet apart, eyes closed, firm base of support;
5. TEC firm—feet tandem, eyes closed, firm base of support;
6. TEO foam—feet tandem, eyes open, standing on foam;
7. FAEC foam— feet apart, eyes closed, standing on foam;
8. TEC foam—feet tandem, eyes closed, with feet on foam.

In the first test, no factors are altered, and it just involves quiet standing. In positions 2, 3, and 4, only one factor is altered: either base of support, vision, or surface compliance. The next four positions vary two factors in each test: either base of support, vision, or surface compliance, taken two at a time. Finally, the eighth test varies all three factors at once, making it very difficult [34].

#### 2.3.5. Postural Sway and Tremor

The balance platform was 1 m by 1 m in size and 0.1 m in height. Four stainless steel bars, each with four strain gauges, were mounted at the four corners under the platform (TML Strain Gauge FLA-6, 350-17, Tokyo, Japan). The output of the four Wheatstone strain gauge bridges was amplified by a Biopac MP35 bio-potential amplifier and digitized through a 24-bit A/D converter. The sampling rate was 2000 samples per second. The sensors were right front (RF), left front (LF), right rear (RR), and left rear (LR).

To calculate the load and the center of the pressure of the force on the platform, the output of the four sensors was used to measure the X and Y coordinates of the center of gravity of the subject. To calculate the movement of the center of pressure from the center of the platform, a series of equations was solved in real time from raw platform data. Diagonal movement toward the RR of the platform would be movement in the first quadrant, movement to the RF of the platform would correspond to movement in the second quadrant, movement to the LF of the platform would be to the third quadrant, and movement to the LR of the platform would be to the fourth quadrant. Movement directly toward the back of the platform would be movement in the +Y direction and movement to the right would be in the +X direction.

The equations and the conditionals accomplished here were solved in real time for each A/D conversion. Thus, the equations were solved 2000 times per second for a continuous presentation of the angle and magnitude of the vector associated with any sway linked to either tremor or movement in the body. These data were converted to a movement vector, giving magnitude and angular displacement. By averaging this movement vector over 2 s, mean and standard deviation (SD) were obtained for this measure. From this, the coefficient of variation (CV) of the polar coordinate was calculated (SD ÷ Mean × 100%) as a measure of the postural sway.

The displacement of the center of pressure (CoP), mean CoP positions, length of the CoP path, sway velocity, area of the CoP path, and root-mean-square area were used to determine postural sway. Some studies have used the coefficient of variation (CV) of the weight displacement as a measure of postural sway [35,36]. Tremor can also be assessed in the 8 Hz band [37] for the variation of movement on the platform [11,38]. The most often encountered tremors have frequencies between 4 and 12 Hz. Tremor in postural deviation usually has a slower frequency of between 3 and 5 Hz, while essential tremor and enhanced physiologic tremor range from 5 to 10 Hz [39,40]. The validity and reliability of this force platform were established in a previous study [41].

#### 2.3.6. Sample Size Estimation

Sample size was determined using G power 3.0.1 (Heinrich-Heine-University Dusseldorf, Dusseldorf, Dusseldorf, Germany). A sample size of 64 participants, with 32 subjects per group, was estimated using an effect size of 0.63 from a previous study [42] with difference between two independent means, level of significance of 0.05, and power of 0.80.

#### 2.3.7. Statistical Analysis

The SPSS 25.0 software for Windows 10 (IBM Corp., Armonk, NY, USA) was used to analyze the data. Data were summarized using the mean and standard deviation (SD). The assumption of normality of the continuous variables was examined using the Shapiro–Wilk test. An independent *t*-test was used to compare general characteristics. Since outcome data were not normally distributed, subjective pain, pressure pain threshold, and postural sway and tremor in each balance task between patients with PF and their controls were compared using the Mann–Whitney U test. A Friedman test was conducted to compare postural sway and tremor in each participant group during eight different balance tasks. The level of significance was set at α = 0.05.

## 3. Results

Plantar fascia thickness was significantly thicker in patients with PF compared to controls (5.9 ± 2.1 vs. 3.6 ± 1.1 mm, *d* = 1.37, *p* < 0.001).

There was a significant difference in mean subjective pain measured with a visual analog scale (VAS) (used 100 mm VAS sheets) between the PF group and the control group (37.6 ± 22.4 vs. 2.1 ± 3.4 mm, *d* = 2.21, *p* < 0.001). PPT was significantly greater in the control group compared to the PF group (28.7 ± 21.4 vs. 2.49 ± 1.34 N, *d* = 1.73, *p* < 0.001).

**Figure 1:**
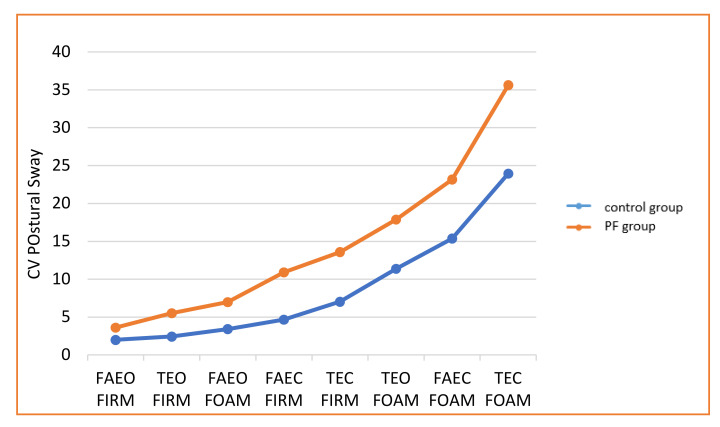
The postural sway in the eight balance task challenges used here by increasing order of difficulty.

**Figure 2:**
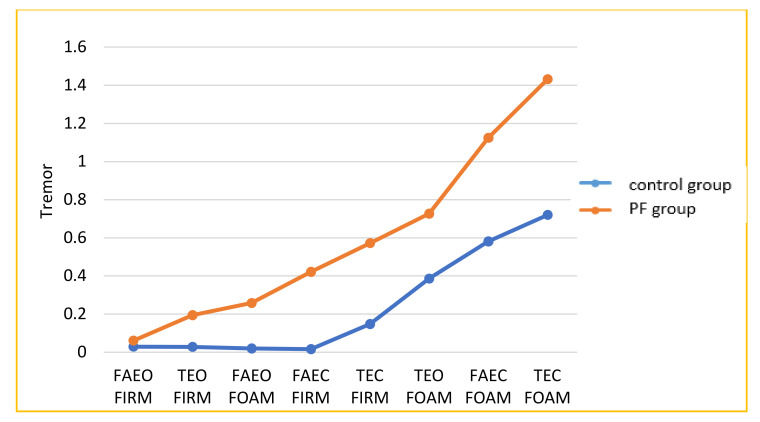
The tremor in the eight balance task challenges used here by increasing order of difficulty.

The results for balance are shown in Figure 1 and Figure 2. The PF group and the controls showed a progressive increase in the CV of sway with increasing complexity of the task. However, the PF group registered significantly higher sway and tremor on all eight tests than the control group (*p* < 0.01, Table 2).

## 4. Discussion

This study tested the hypothesis that the reduction in elasticity associated with PF would reduce balance and increase muscle tremor during balance tasks. The plantar fascia transfers energy from the calcaneus to the toes [43]. Less elasticity would mean that there would be a delay in energy transfer, resembling a spring that has gotten weak. This reduced spring effect would store less energy and also cause greater motor error to take up slack in the plantar fascia, as was seen here with increased tremor. Motor error would also reduce balance, since forward/backward movement at the ankle would be increased in duration. Dorsiflexion of the first metatarsal joint (MTP) is necessary for push-off during ambulation. The MTP joint dorsiflexion begins during heel-off and is at maximum at the toe-off phase of the gait cycle [44]. During this phase of the gait cycle, the hallux is stabilized against the ground, which enables the head of the metatarsal to glide in a plantar and posterior direction on the sesamoids. The MTP joint mechanics described above allow the foot to become a rigid lever, which is necessary for propulsion. Furthermore, the hallux extension places the flexor hallux longus in an optimal length tension to assist the muscle in playing a major role in dynamic balance [45]. In addition, MTP extension transforms the plantar fascia into an important stabilizer, which is responsible for elevation of the medial arch referred to as the “windlass effect” [46,47,48,49]. In addition, tension of the plantar fascia assists the subtalar joint to supinate at push-off. The plantar fascia attaches to the medial process of the calcaneal tuberosity and goes distal to attach to all five digital slips. The plantar fascia serves as an important stabilizer to maintain balance during the “windlass effect”, especially on an unstable surface, as well as working dynamically during foot contact when running. Therefore, the two most important functions of the plantar fascia are assisting the supination of the foot in push-off and the “windlass” mechanism, which is important in maintaining balance. More than any other area of the body, the plantar fascia is a long and complex connection from the muscle, transferring force to the calcaneus and eventually to the toes. The series elastic component in the calf flexors is thus more than simply the tendon to calcaneus, as it also includes the plantar fascia. A reduced elasticity in either the tension or plantar fascia would cause a delay in expressing tension from the calf extensors to the toes. With tension delayed, there would be a delay in time for the spindles to sense the tension and hence increase motor error. Thus, tremor would increase. With higher motor error, it would be harder to balance; therefore, balance would worsen as well.

These results are similar to previous studies on women. In a previous study on women, it was found that during the ovulation phase, because of hormonal changes, there is increased laxity of the plantar fascia, which reduces static and dynamic balance [12]. The hormone causing these changes is estrogen. Estrogen has been shown, through beta estrogen receptors, to cause ligaments and tendons to relax. Thus, in said study, balance and tremor were found to be less pronounced during menstruation and worse during ovulation when estrogen was high [11,15]. In light of these studies, it was relevant to consider the impact of the menstrual cycle on this population for this study. Here, the population was almost one-half women. However, the women here were either postmenopausal or on the birth control pill. Therefore, estrogen levels did not vary. In previous studies, both the birth control pill and menopause were shown to eliminate the normal cyclic variation in hormone circulation seen in women during the menstrual cycle [50,51].

Poor balance has been identified as responsible for increased falls in the elderly [52,53]. In the more active population, overuse injuries of the lower limb are described as inflammatory conditions, such as PF, posterior tibialis tendonitis, Achilles tendonitis and patella tendonitis [33]. In women, these conditions can be caused by the reduced arch height due to possible laxity of the plantar fascia [12]. Could the laxity of the plantar fascia be a factor in the etiology of overuse injuries that are prevalent in women [11]? Can the use of foot orthotics be considered as a treatment approach to assist the function of the plantar fascia in restoring the stability of the medial column of the arch during static and dynamic balance? For example, dynamic balance is important in preventing injuries such as anterior cruciate ligament (ACL) tear. The etiology of ACL tear could be a result of laxity of the plantar fascia.

Like the effect of estrogen in women, edema has been observed to have a similar effect and to cause a similar increase in plantar fascia thickness. It is reasonable that there would be a correlation between plantar fascia edema and falls, but the evidence on this hypothesis is lacking.

The present study has some limitations that need to be addressed. Firstly, a sample size with a large SD might cause a type II error when statistical significance has not been noted. However, the post hoc power analysis using the effect size of 0.76, which was the smallest found in the postural sway and tremor, revealed that the power was 84.2%. Secondly, we did not exclude overweight subjects (BMI ≥ 25 kg/m^2^). The higher BMI could increase plantar fascia thickness, which decreases its stiffness [54]. The average BMI for the present study was 32.6 ± 7.1 kg/m^2^, which might have affected the results of our study. In addition, the subjects in this study were older and generally nonathletes. A younger and more athletic population may provide different results, since age itself reduces balance.

PF is a common clinical condition that reduces gait speed and interferes with the quality of life. The loss in elasticity of the plantar fascia with this condition causes balance to also be poor. The time delay across the foot in developing tension also induces a motor error and increases tremor in the calf muscles. For older patients who may suffer from balance abnormalities already, this makes balance even worse; patients should be cautioned with regard to increased fall risk if this condition continues.

This study involved an older population. It would be interesting to examine younger people and especially women having a normal menstrual cycle vs. women on birth control pills. While static balance was measured here, dynamic balance while walking or running would also be good to measure in the future. Further, the change in plantar elasticity should change impact forces during gait and would also need to be measured during walking and running. Runners affected by PF might be an interesting group to examine to see how their dynamic balance is affected.

## 5. Conclusions

The thickness of the plantar fascia in plantar fasciitis increases from less than 0.4 cm to > 0.4 cm. This swelling not only causes pain but causes a loss in tissue elasticity. With less elasticity, motor error during standing and walking increases as seen by a significant increase in tremor. Motor error also causes impaired balance in this population of people with plantar fasciitis.

## Figures and Tables

**Table 1:** General characteristics of participants (*n* = 50).

Characteristic	Plantar Fasciitis (*n* = 25)	Control (*n* = 25)	*p*-Value
	Mean ± SD	Mean ± SD	
Age (years)	52.1 ± 12.0	49.8 ± 13.9	0.539
Height (cm)	171.3 ± 10.4	166.7 ± 7.2	0.075
Weight (kg)	96.7 ± 20.7	88.9 ± 17.8	0.158
BMI (kg/m^2^)	33.1 ± 7.4	32.1 ± 6.9	0.622

Abbreviations: SD, standard deviation; BMI, body mass index.

**Table 2:** Postural sway and tremor during eight balance tasks in PF group and control group.

	Plantar Fasciitis (*n* = 25)	Control (*n* = 25)	*p*-Value ^a^	Cohen’s d
**Postural Sway**				
FAEO-FIRM	3.60 ± 2.48	1.99 ± 1.63	0.011	0.76
TEO-FIRM	5.52 ± 4.54	2.42 ± 1.13	<0.001	0.93
FAEO-FOAM	6.98 ± 3.83	3.42 ± 1.49	<0.001	1.22
FAEC-FIRM	10.91 ± 8.56	4.67 ± 1.58	<0.001	1.01
TEC-FIRM	13.58 ± 8.01	7.03 ± 2.79	<0.001	1.09
TEO-FOAM	17.88 ± 10.35	11.39 ± 3.69	0.008	0.84
FAEC-FOAM	23.18 ± 13.02	15.38 ± 5.45	0.003	0.78
TEC-FOAM	35.61 ± 15.19	23.94 ± 8.95	0.002	0.94
*p*-Value ^b^	<0.001	<0.001		
**8 Hz Tremor**				
FAEO-FIRM	0.06 ± 0.04	0.02 ± 0.05	<0.001	0.88
TEO-FIRM	0.19 ± 0.16	0.03 ± 0.08	<0.001	1.26
FAEO-FOAM	0.26 ± 0.33	0.04 ± 0.06	<0.001	0.93
FAEC-FIRM	0.42 ± 0.52	0.06 ± 0.03	<0.001	0.97
TEC-FIRM	0.57 ± 0.48	0.15 ± 0.44	<0.001	0.91
TEO-FOAM	0.73 ± 0.55	0.39 ± 0.24	<0.001	0.80
FAEC-FOAM	1.12 ± 0.60	0.59 ± 0.55	<0.001	0.92
TEC-FOAM	1.43 ± 0.45	0.72 ± 0.85	<0.001	1.04
*p*-Value ^b^	<0.001	<0.001		

Abbreviations: SD, standard deviation; BMI, body mass index. ^a^ *p* value from Mann–Whitney U test. ^b^ Friedman test.
