# Erratum: Petrofsky, J., et al. Greater Postural Sway and Tremor during Balance Tasks in Patients with Plantar Fasciitis Compared to Age-Matched Controls. *Healthcare* 2020, *8*, 219

**DOI:** 10.3390/healthcare8040510

**Published:** 2020-11-23

**Authors:** Jerrold Petrofsky, Robert Donatelli, Michael Laymon, Haneul Lee

**Affiliations:** 1 School of Physical Therapy, Touro University Nevada, Henderson, NV 89002, USA; Jerrold.Petrofsky@tun.touro.edu (J.P.); Michael.Laymon@tun.touro.edu (M.L.); 2 Modern Athletic Science, Las Vegas, NV 88901, USA; bobbyd1950@gmail.com; 3 Department of Physical Therapy, Gachon University, Incheon 21936, Korea

Some details of the author affiliations should be corrected in the article [1]. We sincerely apologize to the editor, all editorial staff, the publisher and our readers for any inconvenience.

Haneul Lee is currently affiliated only to the Department of Physical Therapy, Gachon University, Incheon 21936, Korea.

The corrected author list is provided below:

**Jerrold Petrofsky ^1^** **, Robert Donatelli ^2^, Michael Laymon ^1^** and Haneul Lee ^3,^*

^1^ School of Physical Therapy, Touro University Nevada, Henderson, NV 89002, USA; Jerrold.Petrofsky@tun.touro.edu (J.P.); Michael.Laymon@tun.touro.edu (M.L.)

^2^ Modern Athletic Science, Las Vegas, NV 88901, USA; bobbyd1950@gmail.com

^3^ Department of Physical Therapy, Gachon University, Incheon 21936, Korea

* Correspondence: leehaneul84@gachon.ac.kr; Tel.: +82-32-820-4335
